# Biochemical and clinical characteristics of patients with primary aldosteronism: Single centre experience

**DOI:** 10.2478/jomb-2019-0035

**Published:** 2020-01-23

**Authors:** Nataša Vujačić, Ivan Paunović, Aleksandar Diklić, Vladan Živaljević, Nikola Slijepčević, Nevena Kalezić, Mirjana Stojković, Miloš Stojanović, Biljana Beleslin, Miloš Žarković, Jasmina Ćirić

**Affiliations:** 1 Clinical Center of Serbia, Clinic for Endocrinology, Diabetes and Metabolic Diseases, Belgrade; 2 Clinical Center of Serbia, Center of Endocrine Surgery, Belgrade; 3 Clinical Center of Serbia, Clinic of Anesthesiology and Reanimation, Belgrade

**Keywords:** primary aldosteronism, biochemical parameters, clinical presentation, adrenal tumour, hypertension, Cushing syndrome, Cushing-ov sindrom, hipertenzija, adrenalni tumor, klinička prezentacija, biohemijski parametri, primarni aldosteronizam

## Abstract

**Background:**

Primary aldosteronism (PA) is associated with increased prevalence of metabolic disorders (impaired glucose and lipid metabolism and insulin resistance), but also with more frequent cardiovascular, renal and central nervous system complications.

**Methods:**

Biochemical and clinical parameters were retrospectively analysed for 40 patients with PA caused by aldosterone-producing adenoma (APA) and compared to the control groups of 40 patients with nonfunctioning adrenal adenoma (NFA) and essential hypertension (HT), and 20 patients with adrenal Cushing syndrome (CS) or subclinical CS (SCS).

**Results:**

Systolic, diastolic and mean arterial blood pressures were significantly higher in the PA group (p=0.004; p=0.002; p=0.001, respectively) than in NFA+HT group. PA patients had longer hypertension history (p=0.001) than patients with hypercorticism and all had hypokalaemia. This group showed the smallest mean tumour diameter (p<0.001). The metabolic syndrome was significantly less common in the PA group (37.5% vs. 70% in CS+SCS and 65% in NFA+HT group; p=0.015), although there was no significant difference in any of the analysed metabolic parameters between groups. PA group was found to have the most patients with glucose intolerance (81.8%), although the difference was not significant. The mean BMI for all three groups was in the overweight range. Patients with PA had higher microalbuminuria and a higher tendency for cardiovascular, renal and cerebrovascular events, but the difference was not significant.

**Conclusions:**

Our results support the importance of the early recognition of primary aldosteronism on the bases of clinical presentation, as well as an increased screening intensity.

## Abstract (sr)

**Uvod:**

Primarni aldosteronizam (PA) je praćen povećanom prevalencom metaboličkih poremećaja, (oštećen glukozni i lipidni metabolizam i insulinska rezistencija), ali i češćim kardio vaskularnim, renalnim i komplikacijama centralnog nervnog sistema.

**Metode:**

Analizirane su retrospektivno biohemijske i kliničke karakteristike 40 pacijenata sa PA zbog aldosteron-produkujućeg adenoma (APA) i upoređene sa kontrolnim grupama od 40 pacijenata sa afunkcionim adrenalnim adenomom (NFA) i esencijalnom hipertenzijom (HT), i 20 pacijenata sa adrenalnim Cushing-ovim sindromom (CS) ili subkliničkim CS (SCS).

**Rezultati:**

Sistolni, dijastolni i srednji arterijski pritisci su bili značajno viši u grupi sa PA (p=0,004; p=0,002; p=0,001, redom) nego u grupi NFA+HT. PA pacijenti su imali dužu istoriju hipertenzije (p=0,001) nego pacijenti sa hiperkorticizmom i svi su imali hipokalemiju. Ova grupa je imala najmanji srednji prečnik tumora (p<0,001). Metabolički sindrom je bio značajno ređi u PA grupi (37,5% vs. 70% u CS+SCS i 65% u NFA+HT grupi; p=0,015), mada nije bilo značajne razlike u bilo kom analiziranom metaboličkom parametru među grupama. U PA grupi je nađen najveći procenat pacijenata sa glikoznom intolerancijom (81,8%), mada razlika nije bila značajna. Srednja vrednost BMI za sve tri grupe je bila u rangu prekomerne težine. Pacijenti sa PA su imali veći stepen mikroalbuminurije, veću tendencija ka javljanju kardiovaskularnih, renalnih i cerebrovaskularnih događaja, ali razlika nije bila značajna.

**Zaključak:**

Naši rezultati podržavaju značaj ranog prepoznavanja primarnog aldosteronizma na osnovu kliničke prezentacije, ali i povećanog korišćenja skrininga.

## Introduction

In the last fifteen years, increasing knowledge about numerous negative effects of inappropriately high serum aldosterone has led to a widespread interest in screening the general hypertensive population for primary aldosteronism (PA). Some studies that reported a high PA prevalence among hypertensive subjects (up to 15%) favoured this view [1]. More recent data, however, confirmed that prevalence is most probably closer to 5%, which did not support the proposal for general screening [2] [3]. Anyway, the current indications for screening are still quite reasonable and provide for a great number of idiopathic adrenal hyperplasia (IAH) to be diagnosed; this also identified IAH as the most common cause of PA [2]. In recent years, a trend of diagnosing milder forms of PA due to IAH has been observed, as is confirmed in the largest and most recent study [4]. Clinical picture of aldosterone-producing adenoma (APA) is more specific due to the signs of the frequently present hypokalaemia [5]. Still, it accounts for one third of all PA cases, meaning that surgically correctable aldosteronism is not so frequent among general hypertensive population.

Aldosterone oversecretion is associated with an increased prevalence of metabolic disorders such as impaired glucose and lipid metabolism and insulin resistance, but also with an increased occurrence of cardiovascular, renal and central nervous system complications [6]. Atrial fibrillation is found to be twelve times more frequent in PA than in essential hypertension (EHT), myocardial infarction 6.5 times and cerebrovascular infarction 4.2 times, independent of blood pressure and PA subtypes [7].

The aim of this study was to analyse the biochemical and clinical characteristics of PA patients referred to the largest Centre for Endocrine Surgery in Serbia, and to compare them with those found in groups suffering from hypercorticism due to adrenal adenoma and nonsecretory adrenal tumours with EHT.

## Materials and Methods

This is a retrospective cross-sectional study on biochemical and clinical characteristics of patients with primary aldosteronism referred to the Centre of endocrine surgery, Clinical Centre of Serbia, in the period 2007-2017. The diagnosis of primary aldosteronism was previously confirmed by high aldosterone levels and suppressed plasma renin activity (PRA) and/or by confirmatory tests (saline suppression test and captopril test), according to current guidelines [2]. All except one patient had a unilateral adrenal tumour confirmed by computed tomography (CT) scan. Adrenal venous sampling was performed for lateralisation in the case of bilateral tumours and in some patients with small unilateral tumours. Since adrenal venous sampling was not performed routinely, normalization of serum aldosterone and potassium level, as well as the improvement of hypertension after surgery were additionally taken as a confirmation of PA. Exclusion criteria were a lack of analysed data and incomplete or inadequate testing for PA according to the current guidelines.

The PA group was compared to two control groups, one with hypertensive patients with nonfunctioning adrenal tumours and the other with cortisolsecreting tumours. Patients of all three groups had normal 24-h urinary excretion of catecholamines. Patients with nonsecretory adrenal tumours also had normal serum aldosterone: PRA ratio and adequate cortisol suppression by 1 mg of dexamethasone to less than 50 nmol/L. Hypercorticism was confirmed by standardized endocrine tests for ACTH-independent Cushing syndrome. All patients underwent a routine physical examination and their medical history was taken (duration of hypertension, co-morbidities and antihypertensive medications).

Biochemical assessment of the observed parameters was performed by indirect potentiometry (ISE), spectrophotometry and immunoturbidimetry (Roche Cobas 6000). PRA was measured by RIA for angiotensin I (Cisbio), plasma aldosterone and plasma cortisol by the appropriate RIA (Cisbio) kits. Patients were evaluated when normokaleamic and on normal sodium intake assessed by measurement of 24-h urinary sodium excretion.

For the assessment of metabolic characteristics of PA patients compared to the other two groups of hypertensives with adrenal tumours we used weight, height, BMI, waist circumference (WC), serum glucose, HbA1c, insulin, cholesterol, HDL and LDL cholesterol, triglyceride (TG) and CRP. Total-body lipid accumulation products (LAP) were calculated as: (waist circumference (cm) -65) × (triglyceride concentration (mmol/L)) for men and (waist circumference (cm) -58) × (triglyceride concentration (mmol/L)) for women. For renal function assessment we used serum urea, creatinine, creatinine clearance, microalbuminuria, and proteinuria, estimated glomerular filtration rate (eGFR), ultrasound (US) and CT kidney appearance. Chronic renal failure is defined as GFR <60 mL/min/1.73 m^2^ for >3 months. For cardiovascular assessment, we analysed the highest clinical blood pressure on presentation for PA diagnostic tests, cardiovascular events and standard heart US. Blood pressure (BP) was measured by trained personal after at least 5 minutes of rest in a relaxed sitting position, using the auscultatory method with a manual sphygmomanometer. Multiple BP measurements were done, and the average of two readings in the arm with the higher BP was used for final BP value. Metabolic syndrome was defined as the presence of central obesity with WC ≥80 cm for females and ≥94 cm for males plus any two of the following factors: [1] treatment of previously diagnosed hypertension [2] hypertriglyceridemia defined as TG ≥1.7 mmol/L; [3] reduced serum HDL ≤1.03 mmol/L in males and ≤1.29 mmol/L in females; [4] fasting plasma glucose ≥5.6 mmol/L (IDF consensus) [8].

The study was approved by the Ethical Committee of the School of Medicine, University of Belgrade.

Arithmetic mean and standard deviation were used to describe normally distributed numerical data, while median and interquartile range were used for other data. Normal distribution was examined by mathematical and graphical methods. Categorical data were presented by absolute and relative numbers. One Way ANOVA and Kruskal-Wallis tests were used to test the difference in numerical variables between the three study groups. Categorical data were compared by Chi-square test contingency table, or Fisher's exact test, when necessary. After One Way ANOVA test data were analysed by Tukey posthoc test, and after Kruskal-Wallis test Mann Whitney test was applied. The chosen level of confidence for statistical significance for all statistical methods was α=0.05. Complete statistical analysis was done in IBM SPSS ver. 21.0.

## Results

Our study included 40 patients with primary aldosteronism (PA group) and two control groups, the first comprising 40 patients with nonfunctional adrenal adenoma (NFA) and essential hypertension (HT), and the second group consisting of 20 patients with hypercorticism due to adrenocortical tumour, 10 with Cushing's syndrome (CS) and 10 with subclinical CS (SCS). Baseline parameters, including those related to hypertension, are presented in Table 1.

**Table 1:** Baseline characteristics *for the level of significance of 0.05 §Student t test ₤Chi-square test ¥Mann Whitney test SBP=systolic blood pressure, DBP=diastolic blood pressure, MAP= mean arterial pressure, HT=hypertension, HR= heart rate PA vs. NFA+HT: SBP p=0.004; DBP p=0.002; MAP p=0.001; HT duration p=0.120. PA vs. CS+SCS: SBP p=0.127; DBP p=0.114; MAP p=0.080; HT duration 0.001. NFA+HT vs. CS+SCS: SBP p=0.751; DBP p=0.659; MAP p=0.654; HT duration p=0.205.

Characteristic	Total	Groups			p*
		PA	NFA+HT	CS+SCS	
Age, year mean (SD)	52.86 (11.79)	50.50 (11.93)	55.60 (10.54)	52.10 (13.29)	0.147§
Gender, n (%) male female	35 (35.0) 65 (65.0)	15 (37.5) 25 (62.5)	17 (42.5) 23 (57.5)	3 (15.0) 17 (85.0)	0.099£
SBP (mm Hg) mean (SD)	185.35 (27.74)	196.00 (23.51)	176.50 (23.32)	181.75 (36.61)	0.005§
DBP (mm Hg) mean (SD)	109.60 (17.65)	116.75 (19.24)	103.50 (13.83)	107.50 (16.74)	0.002§
Pulse pressure (mm Hg) mean (SD)	75.75 (19.72)	79.25 (19.10)	73.00 (15.88)	74.25 (26.77)	0.344§
MAP (mm Hg) mean (SD)	134.85 (19.43)	143.17 (18.70)	127.83 (15.90)	132.25 (21.78)	0.001§
HT duration (months) med (Q1 – Q3)	96 (48–171)	120 (84–180)	96 (15 –189)	60 (18 –114)	0.010¥
HR (min) mean (SD)	73.99 (9.63)	74.05 (11.09)	73.60 (8.27)	74.65 (9.44)	0.924§

There was no significant difference between groups in age (p=0.147), or sex distribution, although a higher number of women was recorded in all groups (p=0.099). The highest mean values of systolic, diastolic and mean arterial pressure (MAP) were found in the PA group (p=0.005, p=0.002 and p=0.001, respectively). Comparing groups, the difference was statistically significant for systolic (p=0.004), diastolic (p=0.002) and mean arterial pressure (p=0.001) only between the PA and NFA+HT groups. There was no significant difference in pulse pressure (SBP-DBP) value (p=0.344) and heart rate (p=0.924) between groups. The longest hypertension duration was recorded in the PA group, and was significantly longer (p=0.001) than in CS+SCS group.

The largest tumour size was measured in the CS+SCS group, followed by the NFA+HT, while the smallest mean tumour diameter was found in the PA group (p<0.001). Tumour characteristics are presented in Table 2. Serum aldosterone was significantly higher and PRA significantly lower in the PA group (p<0.001), as expected. Similarly, serum cortisol after 1mg of dexamethasone was significantly higher in the CS+SCS group (p<0.001). Two patients with PA had mixed aldosterone and cortisol secretion, PA and SCS based on inadequate cortisol suppression by dexamethasone and increased midnight cortisol levels. Their tumours were larger than those usually seen in aldosteronism, 62×41 mm and 41×33 mm in diameter, respectively. Statistical significance of all compared parameters, especially those related to tumour size and hormone secretion did not change by exclusion of those two patients, so their clinical and metabolic characteristics were also analysed within the PA group. Left-sided hypersecretion of both aldosterone and cortisol were found more frequently, but the difference was not significant. After the adrenal tumour was diagnosed, patients with CS+SCS completed the diagnostic procedure and surgical treatment in the shortest period of time, but the waiting period was significantly shorter only compared to the NFA+HT group (p=0.004). Patients with non-secretory tumours were treated surgically, depending of the tumour size. Significantly smaller number of patients in this group were treated by surgery (p<0.001) compared to the two other groups. Five patients with PA were not surgically treated due to comorbidities, lack of reliable lateralization of aldosterone hypersecretion or personal choice. Hypokalaemia and HT were successfully controlled by spironolactone and antihypertensive drugs. All patients with PA had low serum potassium level, as did 3 patients in both CS and NFA+HT groups. A few patients with PA had a very long history of transient hypokalaemia before the diagnosis and surgery.

**Table 2:** Tumour characteristics *for the level of significance of 0.05 §Student t test ₤Chi-square test ¥Mann Whitney test PA vs. NFA+HT: size p=0.044; aldosterone p<0.001; cortisol p=0.084; PRA p<0.001; K p<0.001; presence of adrenal tumour before surgery p=0.635; operation p<0.001. PA vs. CS+SCS: size p<0.001; aldosterone p<0.001; cortisol p<0.001; PRA p<0.001; K p<0.001; presence of adrenal tumour before surgery p=0.113; operation p=0.825. NFA+HT vs. CS+SCS: size p<0.001; aldosterone p=0.094; cortisol p<0.001; PRA p=0.574; K p=0.760; presence of adrenal tumour before surgery p=0.004; operation p<0.001

Tumour mean (SD) or med (Q1 – Q3)	Total	Groups			p*
		PA	NFA+HT	CS+SCS	
Size, mm	28.98 (13.42)	22.15 (9.70)	27.92 (9.52)	44.75 (14.04)	<0.0005§
Side, n (%)					
Left	54 (54.0)	24 (60.0)	19 (47.5)	11 (55.0)	0.073₤
Right	39 (39.0)	15 (37.5)	19 (47.5)	5 (25.0)	
Bilateral	7 (7.0)	1 (2.5)	2 (5.0)	4 (25.0)	
Aldosterone (ng/L)	307.5 (134.0–808.0)	878.65 (743.87–1225.25)	151.35 (93.0–239)	245.1 (128.5–329.7)	<0.0005¥
Cortisol after 1mg of Dex (nmol/L)	44.5 (31.7–80.9)	42.75 (34.4–51.7)	35.60 (22.5–48.0)	418.30 (211.9–629)	<0.0005¥
PRA (ng/mL/h)	0.49 (0.275–1.685)	0.28 (0.2–0.39)	1.35 (0.5–2.37)	1.57 (0.57–2.89)	<0.0005¥
K (mmol/L)	3.48 (1.09)	2.41 (0.77)	4.24 (0.51)	4.11 (0.60)	<0.0005§
Duration of hypokalaemia (months)	12 (6–39)	12 (6–48)	6 (6–15)	6 (4.5–9)	0.391¥
Presence of adrenal tumour before surgery (months)	12 (4–24)	12 (3–36)	12 (6–24)	7 (3.25-12)	0.042¥
Operation, n (%)					
Yes	57 (57.0)	35 (87.5)	3 (7.5)	19 (95.0)	<0.0005₤
No	43 (43.0)	5 (12.5)	37 (92.5)	1 SCS (5.0)	

Metabolic characteristics for all three groups are shown in Table 3. There was no statistical difference in any of analysed metabolic parameters between groups. The mean BMI for all three groups fell within the overweight range. The highest percent of patients with glucose intolerance was found in the PA group (81.8%), although the difference was not significant.

**Table 3:** Metabolic characteristics *for the level of significance of 0.05 §Student t test ₤Chi-square test ¥Mann Whitney test BMI=body mass index, LAP=lipid accumulation products, LDL=low density lipoprotein, HDL=high density lipoprotein, CRP=C reactive protein Glucose metabolism disorders: PA vs. NFA+HT p=0.431; PA vs. CS+SCS p=0.175; NFA+HT vs. CS+SCS p=0.043.

Characteristic mean (SD) or med (Q1 – Q3)	Total	Groups			p*
		PA	NFA+HT	CS+SCS	
Weight (kg)	80.61 (17.63)	79.88 (15.86)	83.57 (20.57)	75.53 (12.26)	0.255§
Height (cm)	167.02 (13.86)	169.65 (9.81)	165.81 (18.41)	165.4 (25.64)	0.446§
BMI (kg/m^2^)	28.07 (4.48)	27.72 (4.57)	28.53 (4.04)	27.68 (5.30)	0.690§
Waist (cm)	92.88 (13.47)	90.73 (10.61)	94.72 (13.95)	92.37 (16.38)	0.468§
Glycaemia (mmol/L)	5.33 (1.11)	5.32 (1.18)	5.25 (1.02)	5.48 (1.17)	0.745§
Glucose metabolism disorders, n (%)					
Diabetes mellitus	11 (39.3)	2 (18.2)	4 (50.0)	5 (55.6)	0.179₤
Glucose intolerance	17 (60.7)	9 (81.8)	4 (50.0)	4 (44.4)	
Cholesterol (mmol/L)	5.94 (1.22)	5.65 (1.11)	6.04 (1.12)	6.20 (1.51)	0.460§
LDL-cholesterol (mmol/L)	3.78 (1.03)	3.62 (0.91)	3.91 (0.97)	3.83 (1.32)	0.547§
HDL-cholesterol (mmol/L)	1.37 (0.39)	1.34 (0.38)	1.32 (0.39)	1.51 (0.39)	0.166§
TG (mmol/L)	1.80 (1.2–2.2)	1.60 (1–2.2)	1.70 (1.22–2.07)	2 (1.05–2.45)	0.310¥
LAP	48.10 (28–82.8)	41.6 (23.4–75.25)	52.75 (34.5–83.9)	55.1 (27.2–90.2)	0.638¥
CRP (mg/L)	2.10 (1.37–4.55)	2 (1.35–4.9)	2.1 (1.02–4.0)	2.78 (1.5–3.4)	0.777¥

Metabolic syndrome was present in 55% of all adrenal tumour cases. In patients with PA, metabolic syndrome was significantly less common (37.5%, p=0.015). It was most common (70%) in the CS+SCS group (PA vs. CS+SCS p=0.018), followed by nonfunctional adrenal tumour and HT group (65%) (PA vs. NFA+HT p=0.014, and NFA+HT vs. CS+SCS p=0.699).

Renal function parameters are shown in Table 4. Groups differed only in mean serum creatinine levels: the lowest was in the CS+SCS group (p=0.027). The differences between the groups were the following: PA vs. CS+SCS p= 0.031, NFA+HT vs. CS+SCS p=0.045 and PA vs. NFA+HT p=0.982. Other renal function parameters did not differ statistically significantly. A trend of a higher degree of microalbuminuria was observed in the PA group; the difference was significant only comparing to the CS+SCS group (p=0.028). The percent of patients with renal cysts in the PA group was two times higher than in other groups, but this difference reached statistical significance only between PA and NFA+HT groups (p=0.048).

**Table 4:** Renal function parameters *for the level of significance of 0.05 §Student t test Chi-square test ¥Mann Whitney test Creatinine: PA vs. NFA+HT p=0.982; PA vs. CS+SCS p=0.031. NFA+HT vs. CS+SCS p=0.045; Microalbuminuria: PA vs. NFA+HT p=0.335; PA vs. CS+SCS p=0.028; NFA+HT vs. CS+SCS p=0.066.Renal cysts: PA vs. NFA+HT p=0.048; PA vs. CS+SCS p=0.188; NFA+HT vs. CS+SCS p=0.788.

Parameter mean (SD) or med (Q1 – Q3)	Total	Groups			p*
		PA	NFA+HT	CS+SCS	
Urea (mmol/L)	5.37 (1.71)	5.33 (1.91)	5.37 (1.60)	5.44 (1.58)	0.973§
Creatinine (μmol/L)	78.05 (24.96)	81.87 (31.70)	80.87 (16.08)	64.75 (20.47)	0.027§
Clearance Cr (mL/min/1.73 m^2^)	89.65 (25.25)	91.38 (29.33)	86.77 (20.42)	92.01 (26.10)	0.650§
Proteinuria (mg/24h)	0.116 (0.049–0.170)	0.161 (0.032–0.330)	0.084 (0.046–0.133)	0.124 (0.100–0.143)	0.107¥
Microalbuminuria (mg/24h)	0.030 (0.005–0.096)	0.041 (0.018–0.122)	0.009 (0.005–0.076)	0.004 (0.003–0.006)	0.060¥
Renal cysts, n (%)					
yes	20 (20.0)	12 (30.8)	5 (12.5)	3 (15.0)	0.105₤
no	79 (79.0)	27 (69.2)	35 (87.5)	17 (85.0)	

We analysed the presence of major cardiovascular events and renal failure in each group of hypertensive patients, but there was no statistically significant difference between them. There was a greater tendency towards atrial fibrillation in patients with PA compared to the two remaining groups (PA 15%, NFA+HT 5%, CS+SCS 0% respectively, p=0.087). A slightly more frequent appearance of cerebrovascular insult (PA 7.5%, NFA+HT 2.5%, CS+SCS 5% respectively, p=0.591) and acute myocardial infarction (PA 7.5%, NFA+HT 5%, CS+SCS 0% respectively, p=0.454) was observed in the PA group. Chronic renal failure was quite common in all three groups of hypertensive patients (PA 20%, NFA+HT 17.5%, CS+SCS 15% respectively, p=0.808). Other analysed parameters such as angina pectoris, chronic heart failure and aortic aneurysm did not show any specific distribution in groups.

Although all patients with PA were hypokalaemic, many of them had no symptoms. The most frequent symptoms were headache [11], weakness [9] and fatigue [5], which predominantly appeared in females younger than 45 years (14 patients). Women also more often suffered from muscle weakness, numbness and muscle cramps [7] compared to men [2]. Nocturia [3] was reported only by males. Less frequently reported were vomiting [3] and encephalopathy [1]. Associated cardiovascular diseases were all present in patients older than 45 years in the PA group (22 patients). There were multiple cerebrovascular insults among those patients and one male patient experienced acute heart failure. Except for an elderly woman with acute respiratory arrest due to severe hypokalaemia, all hypokalaemic quadriparesis occurrences (4 cases) were in younger patients. Three pregnancies occurred between the time of PA diagnosis and surgical treatment; all pregnancies terminated by caesarean sections due to preeclampsia (27-38 weeks). Premature deliveries resulted in stillborn twins and 2 live newborns.

## Discussion

Hypertension in primary aldosteronism is generally described as mild-to-moderate and resistant to the treatment. According to the study by Mosso et al. [9], among 600 hypertensive patients screened for primary aldosteronism, the diagnosis was confirmed in 2% of patients with mild hypertension, 8% with moderate and in 13% of patients with severe hypertension. These results suggest that the prevalence of PA increases with severity of hypertension. The exact percentage of severe hypertension across the large spectrum of PA is less clear, but many sporadic cases of hypertensive emergencies from the literature demonstrate the importance of its early recognition [10]. In addition, sustained increase of aldosterone secretion may cause significant and irreversible vascular damage, leading to residual hypertension after surgical removal of the aldosterone-producing adenoma [11].

Severe hypertension is not specific only for aldosterone overactivity. Still, in this study we found the highest systolic, diastolic and mean blood pressures in the PA group of hypertensives compared to groups of patients with cortisol-secreting tumours and especially with EHT and adrenal tumours. Study of Mosso et al. [9] was one among many studies that confirmed higher blood pressure in PA than in EHT. The prevalence of hypertension in CS is about 80% and it correlates significantly with the duration of hypercortisolaemia [12]. In CS, the prevalence of hypertension and blood pressure level depend on the cause or the degree of hypercortisolism, and in rare cases, on co-secretion of other adrenal steroids. Severe hypertension has been reported in 17% of cases; in the remaining cases it is rather mild-to-moderate [12]. Hypercortisolaemia may have a profound effect on hypertension through several mechanisms. Among the most important are mineralocorticoid (MK) effects of cortisol via MK receptors, activation of the renin-angiotensin system and effects on peripheral and systemic vasculature [13].

Analysing the period from the detection of the adrenal tumour and/or hypokalaemia to the surgical treatment, the shortest period in our study was observed in the group of patients with CS and even SCS. Clinical picture of CS facilitates its early recognition. In patients with subclinical form of CS, the level of cortisol is lower throughout the day than in the clinically apparent form and it has a smaller effect on hypertension severity. Still, more frequent use of screening for hypercorticism (the dexamethasone test) than for primary aldosteronism (the aldosterone : PRA ratio) in patients with adrenal tumours, as well as better understanding of the adverse effects of cortisol than aldosterone may contribute to more efficient diagnosis and treatment of patients with SCS than those with PA. This finding may be particularly disappointing since we have considered only patients with APA, known to have more severe hypertension, more frequent hypokalaemia, and higher plasma and urinary aldosterone levels than those with IAH [2]. In a recent study on nearly 37000 patients with hypertension and hypokalaemia, only 2.7% were ever screened for PA, confirming that the insufficient screening of at-risk hypertensives could be a universal problem [14].

The hypertension duration was the longest in the PA group with a maximum of 26 years. Insidious onset of PA with hypokalaemia and worsening of hypertension in many of our patients suggest the need for constant monitoring of hypertensive individuals. The unfavourable delay of PA diagnosis is caused by many specific characteristic of PA, such as small tumour size, frequently invisible on ultrasound scan, complex and ambiguous diagnostics, inability to lateralize the aldosterone hypersecretion and comorbidities. Also, people unwilling or unable to complete preoperative testing and to proceed with surgical treatment have a good option to be treated with an MR antagonist. Treatment with spironolactone was shown to be almost equally as effective in disease control as surgery [15] [16].

Aldosterone-secreting tumour is generally small, usually < 2 cm in diameter on CT [2]. The mean diameter found in our study was 22 mm, significantly smaller than in the remaining two groups, even though two patients with large tumours and mixed cortisol and aldosterone secretion were also included. Both patients had very high levels of serum aldosterone, symptoms of severe hypokalaemia and lack of any sign of cortisol hypersecretion (SCS). Cortisol-cosecreting type of APA is increasingly recognized in the last fifteen years and mostly presented as an adenoma larger than 2.5 cm [17]. The tumours seen in our two patients were among the largest reported to date (62x41 mm, 76 g). According to a recent multicentre study, steroid metabolome analysis revealed that patients with PA frequently have significantly increased cortisol and total glucocorticoid metabolite excretion [18]. The authors found that glucocorticoid excretion *in vivo* showed a statistically significant association with the corresponding intratumoral CYP11B1 expression and significantly correlated with metabolic risk parameters [18]. We did not find any statistical difference in the analysed metabolic parameters between groups in our study. Still, subclinical and clinically overt adrenal Cushing syndrome as well as NFA, found incidentally or due to hypertension, are known to be associated with unfavourable metabolic profile [19] [20]. The mean BMI for all three groups was in the overweight range. The highest percent of patients with glucose intolerance was found in the PA group (81.8%), although the difference was not significant. Metabolic syndrome was found significantly less commonly in the PA group than in the remaining two groups, but the overall appearance (37%) is still considerable. The prevalence of metabolic syndrome in the general population varies, being higher in older and overweight sedentary subjects. Ribeiro Cavalari et al. [21] showed that the frequency of prediabetes, dyslipidaemia, increased waist circumference, and hypertension are significantly higher in the group of patients with nonfunctioning adrenal tumours compared to matched controls with normal adrenal imaging. Using different criteria for definition, they also demonstrated that NFA patients have a high prevalence of metabolic syndrome, significantly higher than the control group (according to IDF: 78.6% vs. 45.5%; P < 0.001). In line with the majority of current criteria for metabolic syndrome definition used in their study, after logistic regression analysis, the presence of NFA was shown to be a good predictor of its appearance [21]. In their study on metabolic syndrome prevalence in PA, Somlóová et al. [22] showed that metabolic profile of patients with bilateral form of PA (IHA) is similar to EHT in contrast to the unilateral form of PA (APA). The prevalences of the metabolic syndrome in their study were 62% in IHA, 34% in APA and 56% in EHT. Insulin resistance is believed to play a role in the pathogenesis of NFA, directly through the mitogenic effect on the adrenal cortex or through a subtle increase of cortisol secretion [23]. The opposite causal relationship is also possible, and such a complex relationship of mutual stimulation is also demonstrated between insulin resistance and the low grade hypersecretion of aldo sterone in *zona glomerulosa* in overweight/obese and normo-or hypertensive subjects [24] [25] [26].

Cardiovascular and renal damage in PA generally exceed the effects of high blood pressure. Some small studies such as Catena et al. [27], found a 24-h creatinine clearance of <60 mL/min/1.73 m^2^ in 7% of 56 patients with PA. In a large German study plasma creatinine levels were elevated in 29%, significantly higher than in hypertensive controls (10%) [28]. Also, regression analysis showed that age, male sex, low potassium, and high aldosterone concentrations were independent predictors of lower GFR. In our study, 20% of patients in the PA group had lower than normal GFR and a tendency towards a higher degree of proteinuria and microalbuminuria compared to other groups. A limitation of our study is the small number of participants, since this is a singlecentre study and APA is significantly less common as the cause of primary aldosteronism than IAH. According to the large PAPY (Primary Aldosteronism Prevalence in Italy) study, 24-h microalbuminuria is significantly greater in subjects with both APA and IHA subtypes than in EHT [29]. The lowest mean serum creatinine level in the group of CS+SCS could be explained by the profound catabolic effect of cortisol, or by the lower extent of renal damage than in PA and EHT.

One of the earliest studies using CT scans for renal assessment in 55 patients with PA showed the presence of renal cysts in 44% of the cases, more frequently in the APA (62%) than in the IAH group [30]. Cysts were often multiple and located in the medulla, with the tendency of healing after tumour removal. Lower plasma potassium and higher serum aldosterone levels were correlated with the extent of the cystic disease. In the study of Novello et al. [31], 54 patients with PA were analysed by renal US before and after adrenalectomy or treatment with aldosterone antagonists. Prevalence and average number of renal cysts were significantly greater in patients with PA than in hypertensive and normotensive controls. They also showed that age and plasma potassium level were independently associated with the presence of renal cysts in PA. In our study, patients in the PA group had renal cysts twice as often as those in NFA+HT and CS+SCS groups. However, the difference was statistically significant only compared to the EHT group, possibly due to the small number of patients.

In addition to hypertension, hypokalaemia may greatly contribute to organ damage in PA. Analysing the group of 553 patients from German Conn's registry, authors found that in hypokalaemic PA (56.1%), systolic and diastolic blood pressures were significantly higher than in those with normokalaemia [32]. The over all prevalence of comorbidities was significantly higher in hypokalaemic PA. However, considered individually, only angina pectoris and chronic cardiac insufficiency were significantly more prevalent in hypokalaemic patients (9.0 vs. 2.1%; and 5.5 vs. 2.1%). They also showed an overall high prevalence of comorbidities in patients with PA; the prevalence of cardiovascular events (angina pectoris, myocardial infarction, chronic cardiac insufficiency, coronary angioplasty) was 16.3%, and atrial fibrillation 7.1%. Cerebrovascular comorbidities did not differ between the groups [32].

In conclusion, this is the first study on biochemical and clinical characteristics of patients with primary aldosteronism in our county. It revealed that patients with aldosterone-producing adenoma have significantly higher systolic, diastolic and mean blood pressures compared to patients with nonfunctioning adrenal adenoma with hypertension. All patients with PA also had hypokalaemia, which aggravated organ damage or hypertensive emergencies. The rate of cardiovascular, renal and cerebrovascular events, microalbuminuria and metabolic profiles did not differ between groups, although the metabolic syndrome was significantly less common in primary aldosteronism.

### Conflict of interest statement

The authors state that they have no conflicts of interest regarding the publication of this article.

## List of abbreviations

PA, primary aldosteronism; IAH, idiopathicadrenal hyperplasia; APA, aldosterone-producing adenoma; EHT, essential hypertension; PRA, plasma renin activity; ACTH, adrenocorticotropic hormone; BMI, body mass index; WC, waist circumference; CRP – c reactive protein; SBP, systolic blood pressure; DBP, diastolic blood pressure; HDL, high-densitylipoprotein cholesterol; LDL, low density lipoprotein cholesterol; TG, triglycerides; LAP, lipid accumulation product; HbA1c, haemoglobin A1c
